# Supplementary material for: Assessing the Test-Retest Reliability of MyotonPRO for Measuring Achilles Tendon Stiffness
Source: J Funct Morphol Kinesiol. 2025 Feb 28;10(1):83. doi: 10.3390/jfmk10010083 (PMC11942912; doi:10.3390/jfmk10010083)

Supplementary Materials

Supplementary Photo S1. MyotonPRO

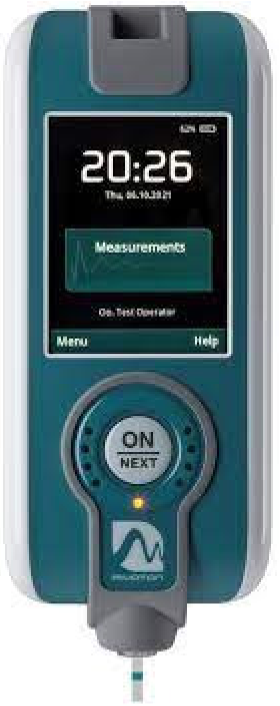

Supplementary Photo S2. Adjustable rack for standardization

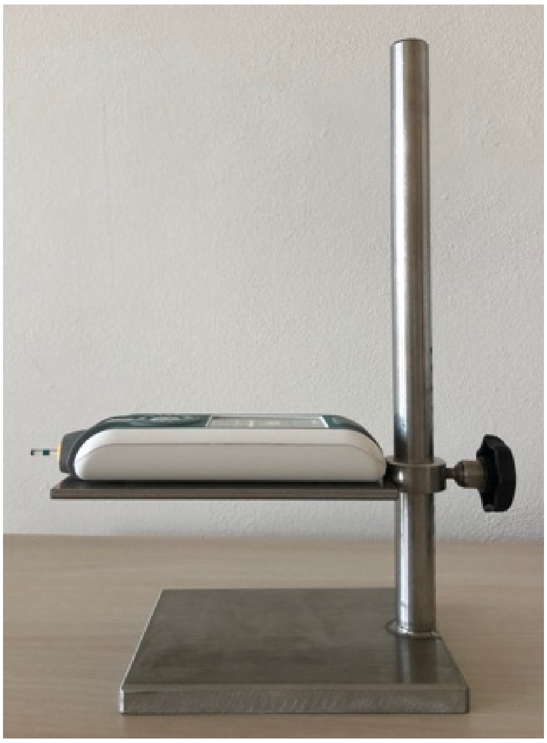

Supplementary Table S1. Sport simulation loading protocol (SP)

Type of contraction	Reps	Sets	Range of motion (°)	Angular velocity (°/s)	Time under tension for one set (s)	Total time under tension (s)	Pause between sets (s)	Total pause (s)
isometric	1	12	0	0	15	180	30	330
eccentric	5	12	45	15	15	180	30	330
concentric/eccentric	10	12	45	60	15	180	30	330

Supplementary – Systemic Bias Analysis

Bland-Altman

Immediate SM – 1. and 2.

Bland-Altman Statistics
======================
t = -3.8831, df = 479, p-value = 0.0001176
alternative hypothesis: true bias is not equal to 0

======================
Number of comparisons: 480
Maximum value for average measures: 1400
Minimum value for average measures: 592
Maximum value for difference in measures: 238
Minimum value for difference in measures: -284

Bias: -5.427083
Standard deviation of bias: 30.61988

Standard error of bias: 1.3976
Standard error for limits of agreement: 2.390157

Bias: -5.427083
Bias- upper 95% CI: -2.680899
Bias- lower 95% CI: -8.173268

Upper limit of agreement: 54.58788
Upper LOA- upper 95% CI: 59.28437
Upper LOA- lower 95% CI: 49.8914

Lower limit of agreement: -65.44205
Lower LOA- upper 95% CI: -60.74556
Lower LOA- lower 95% CI: -70.13854

======================
Derived measures:
Mean of differences/means: -0.6195373
Point estimate of bias as proportion of lowest average: -0.916737
Point estimate of bias as proportion of highest average -0.3876488
Spread of data between lower and upper LoAs: 120.0299
Bias as proportion of LoA spread: -4.521442

======================
Bias:
-5.427083 (-8.173268 to -2.680899)
ULoA:

54.58788 (49.8914 to 59.28437)
LLoA:
-65.44205 (-70.13854 to -60.74556)

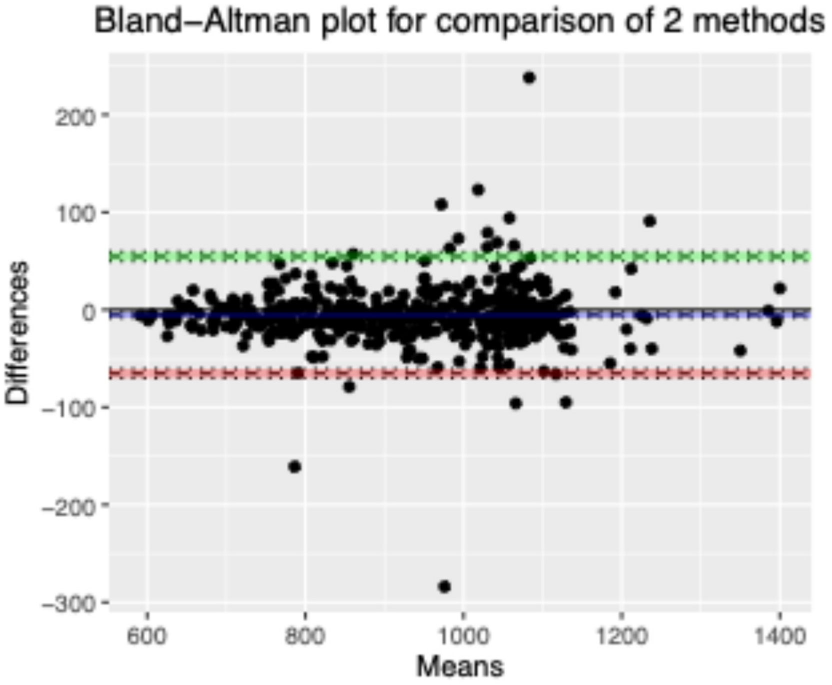

Immediate SM – 2. and 3.

Bland-Altman Statistics
======================
t = -4.1725, df = 479, p-value = 3.579e-05
alternative hypothesis: true bias is not equal to 0

======================
Number of comparisons: 480
Maximum value for average measures: 1403
Minimum value for average measures: 603
Maximum value for difference in measures: 205
Minimum value for difference in measures: -181

Bias: -6.091667
Standard deviation of bias: 31.9863

Standard error of bias: 1.459968
Standard error for limits of agreement: 2.496818

Bias: -6.091667
Bias- upper 95% CI: -3.222933
Bias- lower 95% CI: -8.9604

Upper limit of agreement: 56.60147
Upper LOA- upper 95% CI: 61.50754
Upper LOA- lower 95% CI: 51.6954

Lower limit of agreement: -68.78481
Lower LOA- upper 95% CI: -63.87874
Lower LOA- lower 95% CI: -73.69088

======================

Derived measures:
Mean of differences/means: -0.6265751
Point estimate of bias as proportion of lowest average: -1.010227
Point estimate of bias as proportion of highest average -0.4341886
Spread of data between lower and upper LoAs: 125.3863
Bias as proportion of LoA spread: -4.85832

======================
Bias:
-6.091667 (-8.9604 to -3.222933)
ULoA:
56.60147 (51.6954 to 61.50754)
LLoA:
-68.78481 (-73.69088 to -63.87874)

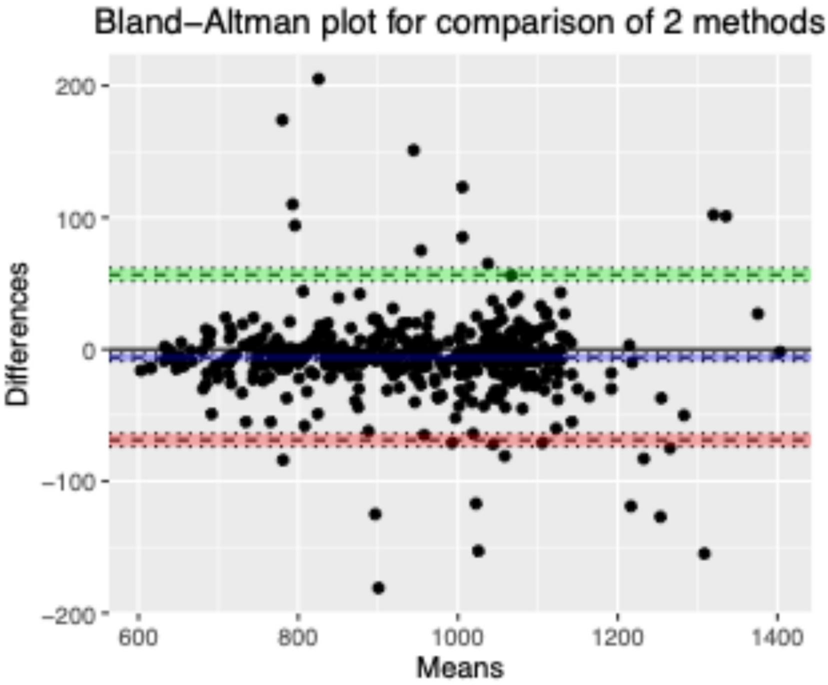

Immediate SM – 1. and 3.

Bland-Altman Statistics
======================
t = -6.6569, df = 479, p-value = 7.678e-11
alternative hypothesis: true bias is not equal to 0

======================
Number of comparisons: 480
Maximum value for average measures: 1397
Minimum value for average measures: 600
Maximum value for difference in measures: 187
Minimum value for difference in measures: -302

Bias: -11.51875
Standard deviation of bias: 37.91028

Standard error of bias: 1.73036
Standard error for limits of agreement: 2.959239

Bias: -11.51875
Bias- upper 95% CI: -8.118716

Bias- lower 95% CI: -14.91878

Upper limit of agreement: 62.78541
Upper LOA- upper 95% CI: 68.6001
Upper LOA- lower 95% CI: 56.97071

Lower limit of agreement: -85.82291
Lower LOA- upper 95% CI: -80.00821
Lower LOA- lower 95% CI: -91.6376

=======================
Derived measures:
Mean of differences/means: -1.245603
Point estimate of bias as proportion of lowest average: -1.919792
Point estimate of bias as proportion of highest average -0.8245347
Spread of data between lower and upper LoAs: 148.6083
Bias as proportion of LoA spread: -7.751081

=======================
Bias:
-11.51875 (-14.91878 to -8.118716)
ULoA:
62.78541 (56.97071 to 68.6001)
LLoA:
-85.82291 (-91.6376 to -80.00821)

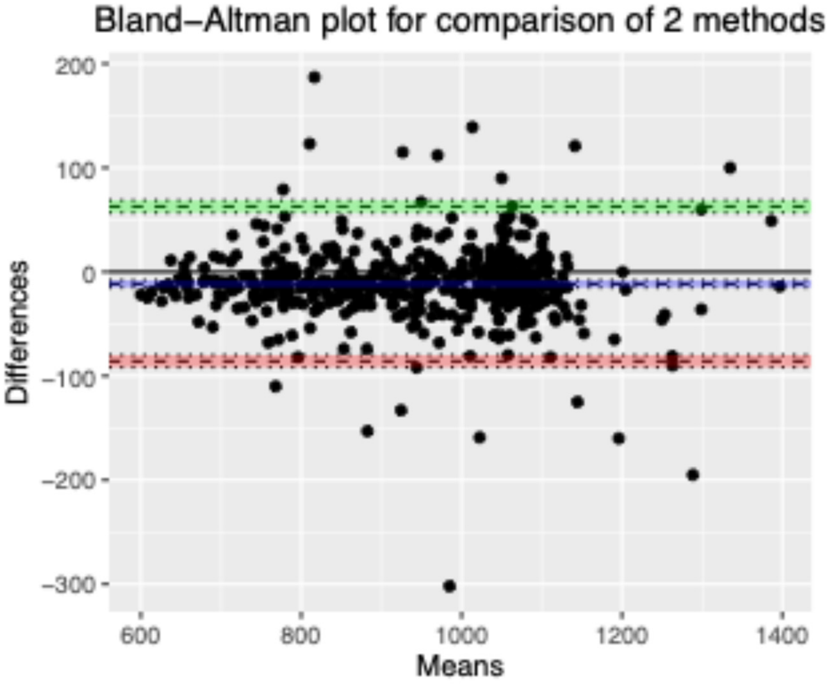

Supplement: Supplementary file 1 [file jfmk-10-00083-s001.zip › jfmk-3411868-supplementary.pdf]
